# Conquering the host: *Bordetella* spp. and *Pseudomonas aeruginosa* molecular regulators in lung infection

**DOI:** 10.3389/fmicb.2022.983149

**Published:** 2022-09-26

**Authors:** Alina M. Holban, Courtney M. Gregoire, Monica C. Gestal

**Affiliations:** ^1^Research Institute of the University of Bucharest (ICUB), Bucharest, Romania; ^2^Department of Microbiology and Immunology, Faculty of Biology, University of Bucharest, Bucharest, Romania; ^3^Department of Microbiology and Immunology, Louisiana State University Health Science Center, Shreveport, LA, United States

**Keywords:** *Bordetella*, *Pseudomonas*, immunosuppression, host-pathogen, stress response, biofilm, virulence

## Abstract

When bacteria sense cues from the host environment, stress responses are activated. Two component systems, sigma factors, small RNAs, ppGpp stringent response, and chaperones start coordinate the expression of virulence factors or immunomodulators to allow bacteria to respond. Although, some of these are well studied, such as the two-component systems, the contribution of other regulators, such as sigma factors or ppGpp, is increasingly gaining attention. *Pseudomonas aeruginosa* is the gold standard pathogen for studying the molecular mechanisms to sense and respond to environmental cues. *Bordetella* spp., on the other hand, is a microbial model for studying host-pathogen interactions at the molecular level. These two pathogens have the ability to colonize the lungs of patients with chronic diseases, suggesting that they have the potential to share a niche and interact. However, the molecular networks that facilitate adaptation of *Bordetella* spp. to cues are unclear. Here, we offer a side-by-side comparison of what is known about these diverse molecular mechanisms that bacteria utilize to counteract host immune responses, while highlighting the relatively unexplored interactions between them.

## Introduction

Respiratory infections caused by bacterial pathogens lead to changes in the structure of the airways and can eventually limit respiratory function. Though viruses and some bacteria, such as *Bordetella pertussis*, initiate an acute “bronchitis” in healthy individuals, a vast number of patients with disrupted lung immunity or mucosal clearance suffer from infections that typically do not resolve with antibiotic treatment (Pragman et al., 2016).

*Haemophilus influenzae* and *Pseudomonas aeruginosa* are the most common pathogens found in the sputum of non-cystic fibrosis bronchiectasis patients. *P. aeruginosa* is also the most frequent opportunistic pathogen associated with severe outcome and high mortality in ventilated and cystic fibrosis patients (Demko et al., 1995). Many *P. aeruginosa* clones that cause severe chronic lung infections are well adapted to their niche and are often unable to grow under standard culture conditions *in vitro* (Penterman et al., 2014; Pragman et al., 2016). Genotypic and phenotypic diversity can affect the microbiome of the airways and the levels of antimicrobial resistance, making it difficult to eradicate the infection (Feltner et al., 2016; Darch et al., 2017; Sønderholm et al., 2017; Azimi et al., 2020). Chronic lungs exhibit biofilm-type infection with *P. aeruginosa* which is accompanied by regular release of planktonic bacteria, possibly due to host factors and/or specific environmental signals (Zegans et al., 2002; Furukawa et al., 2006), allowing the biofilm to spread and grow.

In 1993, Muller and Hildebrandt did phylogenic analysis on the 3 classical *Bordetella* spp. using 23S rRNA analysis. Their results revealed >99% homology between the three classical species of *Bordetella*. Interestingly, the results also revealed certain degree of homology between these *Bordetella* species and *Pseudomonas aeruginosa* (Müller and Hildebrandt, 1993). Both bacteria follow under the Phylum Pseudomonadota, from which then it is divided into Gammaproteobacteria (*Pseudomonas* spp.) or betaproteobacteria (*Bordetella* spp.). *Bordetella* species are most closely related to bacteria classified in the genera *Alcaligenes* (Varley, 1986; Gerlach et al., 2001) and *Achromobacter* (Gross et al., 2008). While *Pseudomonas* spp. are most similar to *Acinetobacter* spp. or *Moraxella* spp. Although *Bordetella* spp. is from the family Alcaligeneaceae (Vandamme et al., 1996; Gerlach et al., 2001), *Alcaligenes* spp. has also genetic and metabolic similarities with *Pseudomonas* spp. (Gross et al., 2008; Brickman and Armstrong, 2018). Both have evolved from environmental origins (Mathee et al., 2008), and both have evolved a variety of virulence mechanisms that allows them to be adaptable to different environmental stressors, including host immune responses (Hauser, 2011; Gestal et al., 2019a,b; Qin et al., 2022). *B. pertussis* is the causative agent of whooping cough, also known as pertussis disease or the 100-day cough (Kilgore et al., 2016). The bacterium is highly transmissible, with an R0 as high as 17 and significant mortality before a vaccine was introduced (Kilgore et al., 2016). Unlike other respiratory pathogens, pertussis may not always show signs of an inflammatory response due to the bacteria’s ability to modulate the host immune responses (Gestal et al., 2018, 2019a,b) or evade them via biofilms (Cattelan et al., 2017). Despite the traditional view of *Bordetella* infection as a pediatric disease, the number of adolescent and adult patients with underlying conditions who are infected with *Bordetella bronchiseptica* (Spilker et al., 2008; Bos et al., 2011; Register et al., 2012; Brady et al., 2014; Sukumar et al., 2014; El Khatib et al., 2015; Menetrey et al., 2021) or non-classical *Bordetella* spp. (Zelazny et al., 2013; Biederman et al., 2015) is increasing, shifting the classical view of these pathogen as exclusive for the infant population (Funke et al., 1996; Coenye et al., 2002; De Schutter et al., 2003; Moissenet et al., 2005; Homola et al., 2012; Karamooz et al., 2018; O’Grady et al., 2019). Importantly, infections with *Bordetella* spp. have three distinct stages, the catarrhal stage, which is characterized by the mucosal discharge; the paroxysmal stage, which associates with the violent and constant coughing; and finally, the pneumonic stage that in infants can lead to death (Kilgore et al., 2016). Although *Bordetella* spp. infection might not 100% match the classical description of chronic infection, it is a long term disease that once over leaves the patients with a number of sequelae (Kilgore et al., 2016).

*Pseudomonas aeruginosa* and *Bordetella* spp. are both respiratory pathogens that require mucosal responses to be cleared from the lungs of infected patients (Blackwood et al., 2020). Recent and exciting results suggest that *P. aeruginosa* and *Bordetella* spp. Interact (Irie et al., 2005), and even share a niche, in the lungs of patients with chronic infections; however, the interface between these two organisms remains unexplored. Both species harbor highly sophisticated mechanisms to manipulate host immune responses and colonize the respiratory tract in similar ways (Blackwood et al., 2020). Therefore, complementary studies using the strengths of each model will bring a better understanding of the complex regulation of bacterial immunomodulatory pathways.

In this review, we contextualize the molecular markers which were recently being related to the infectious process of *P. aeruginosa* and *Bordetella* spp. in the respiratory tract. We have chosen these two pathogens because *P. aeruginosa* is one of the models for the study of quorum sensing (QS) and interkingdom communication, while *Bordetella* spp. is the gold standard model for the study of host-pathogen interaction. Moreover, *Bordetella* spp. is increasingly infecting chronic lung patients such as those suffering from Cystic Fibrosis or COPD (Wallet et al., 2002; Spilker et al., 2008; Menetrey et al., 2022; Moore et al., 2022) and naturally cause infection in cystic fibrosis animal models (Rogers et al., 2008; Darrah et al., 2017), suggesting that *Bordetella* spp. and *Pseudomonas* spp. can share or compete for the same niche. Understanding *Bordetella* spp. and *Pseudomonas spp.* signaling mechanisms, biofilm formation, their pathogenesis in chronic lung disease patients, and their interactions with other microbes is critical as infections produced by these microbes are increasing.

## Host interaction and colonization

*Pseudomonas aeruginosa* and *Bordetella* spp. follow similar steps in the development of lung infection, utilizing well investigated virulence factors responsible for adherence, invasion, and host immune modulation. However, in recent years the roles of multiple molecules involved in the control of microbial virulence during infection is being elucidated. As studies progress, the molecular connections between bacteria and their host, as well as particularities among different bacteria pathogens during infection are offering a wider perspective regarding the complexity of pathogen-pathogen and host-pathogen interactions (Otero-Asman et al., 2020; Sultan et al., 2021; Trouillon et al., 2021). Here we will briefly describe the main mechanisms to suppress host immune response, but our special focus will be on the regulatory pathways that allow to respond to host stressors helping colonization.

### Sensing cues: Two component systems

Once in the host, the first challenge is to sense host-inflammatory cues, and host environment, a successful sensing and adaptation to that new environment will allow pathogens to successfully colonize the host. Bacteria have mechanisms that allow them to sense external cues and respond accordingly to better improve their fitness (Gestal et al., 2019b). To accomplish a successful colonization, bacteria need to express cell-associated virulence factors (i.e., appendages involved in adherence and immune modulation), whose expression occurs after bacteria detect the host environment and inflammatory signals. One of the best studied mechanisms that bacteria utilize to respond to environmental cues are the two component systems (TCS). The simplest TCSs are composed of a protein that senses the external stimuli (e.g., the sensor kinase) that, once phosphorylated, transfers the phosphate group to the effector protein (response regulators), which then regulates gene expression (West and Stock, 2001).

*Pseudomonas aeruginosa* uses its TCS to detect host molecules and respond, and for that they are critical in host-pathogen signaling (Turkina and Vikström, 2019). Host iron, cytokines, and stress hormones are detected by *P. aeruginosa* (Holban et al., 2016; Turkina and Vikström, 2019) and *Bordetella* spp. (Gestal et al., 2019b) via TCS and are used as signaling molecules that regulate bacterial virulence. As a response, bacterial molecules are produced to modulate host phenotypes, involved in inflammatory responses such as chemotaxis, cell migration, phagocytosis, cell differentiation, and apoptosis (Holban et al., 2014a; Turkina and Vikström, 2019). Flagellum, type IV pili, type 3 and type 6 secretion systems (T3SS and T6SS), exopolysaccharides (i.e., alginate), lipopolysaccharides, and secreted proteases (LasA, LasB, AprA, Protease IV) are the traditional virulence factors employed in initial host interaction and colonization by *P. aeruginosa* (Faure et al., 2018). These virulence factors are involved in tissue colonization, invasion, and evasion of the host immune response. The expression of these virulence factors is constantly regulated by the molecular signals detected by the bacteria via TCSs, which are specialized in sensing, responding, and adapting to external cues (Gestal et al., 2019b). *P. aeruginosa* uses multi-kinase networks for sensing and integrating multiple signals to increase bacterial fitness and survival. Over 45 conserved response regulators were identified in the core TCSs regulatory network in *P. aeruginosa* (Trouillon et al., 2021). These TCSs ensure the switch between acute and chronic phases, and modulate phenotypes that are required for infection, including biofilm, motility, and virulence (Preston et al., 1998; Workentine et al., 2009; Ortiz-Martín et al., 2010; Moscoso et al., 2011; Rasamiravaka et al., 2015; Chambonnier et al., 2016; Francis et al., 2017). The main functions targeted by TCS in *P. aeruginosa* include porin activity, DNA-binding, and transcription factor activity (Trouillon et al., 2021). One of the most important TCS in *P. aeruginosa* is the GacS-GacA system, which upregulates small RNAs (sRNAs) involved in polysaccharide, rhamnolipid, and lectin production, extracellular DNA release, QS signaling, iron metabolism (Francis et al., 2017), and motility; all highly involved in host colonization (Pérez-Martínez and Haas, 2011). Other TCS and sRNAs (which will be described in more detail later) that control *P. aeruginosa* virulence include: (1) FleSR, GacSA, CreCB, CarSR, PilSR, FimS-AlgR, ChpA-PilG (which regulates motility and may be involved in the initial host colonization); (2) FleSR, PilSR, RocS1-RocR, RocA1, FimS-AlgR, KinB-AlgB, BfiSR, GacSA, RetS, RcsCB, PvrSR, MifSR, BfmSR, PprAB, BqsSR (which regulates biofilm formation and could impact on the acute/chronic infection switch); and (3) TtsSR, GtrS-GltR, GacS-LadS-RetS, CsrA/RsmA, RsmA, LadS, RocS1-RocR, RocA1, CbrAB, PA2573-PA2572, PhoRB, FimS-AlgR (which controls the expression of soluble enzymes and toxins, which are involved in host invasion) (Sultan et al., 2021).

In *Bordetella* spp., the primary TCS master regulon (*Bordetella* virulence genes, Bvg) (Moon et al., 2017) controls virulence (Coutte et al., 2020). A protein-protein blast of BvgA revealed that it shares 98% homology with *P. aeruginosa* response regulator transcription factors some such as the well-known Lux-family (Venturi, 2006). Some of the genes regulated by Bvg include important virulence factors such as filamentous hemagglutinin (FHA), fimbriae (Fim), and adenylate cyclase toxin (ACT) (Nicholson, 2007; Espinosa-Vinals et al., 2021). But Bvg similar to the Gac system in *P. aeruginosa*, regulates not only virulence factors but also sigma factors (Nicholson et al., 2009; Moon et al., 2017; Coutte et al., 2020) and sRNAs (Moon et al., 2021), which are also regulators, and the molecular mechanisms that interconnect them, remain not fully understood (Gestal et al., 2019b). The Bvg two component systems is not the only TCS in *Bordetella* spp. and our knowledge about *Bordetella* spp. sensory mechanisms is rapidly increasing. Up to date, the other TCS are the RisAS that is important for intracellular survival (Jungnitz et al., 1998; Zimna et al., 2001), and the PlrSR which is critical for colonization of the lower respiratory tract (Bone et al., 2017). Many TCSs in *Bordetella* spp. are not yet characterized, leaving the question of how they interact with each other and orchestrate the response signals unanswered. Understanding the intracellular signaling mechanisms that dictate the responses of *Bordetella* spp. is still in early stages (Gestal et al., 2019b), but we can use the knowledge gained on *P. aeruginosa* as a model to guide us in our investigations as the protein homology between the two component systems of both organisms is remarkable (Table 1).

**TABLE 1:** The most significant TCS of both organisms and their function.

	*Pseudomonas aeruginosa*	*Bordetella* spp.
Virulence	GacS-GacA (Francis et al., 2017), SagS (Petrova and Sauer, 2011), BfiS-BfiR (Petrova and Sauer, 2009), HptB-HsbR (Bhuwan et al., 2012), LadS (Chambonnier et al., 2016), RetS (Moscoso et al., 2011), RocS2-RocA2 (Sivaneson et al., 2011), RocS1–RocR–RocA1 (Kuchma et al., 2005), PvrS-PvrR (Mikkelsen et al., 2013), ChpA/PilG/PilH/ChpB (Inclan et al., 2016), FimS (AlgZ)-AlgR (Okkotsu et al., 2014), PhoQ–PhoP (McPhee et al., 2006), ColS-ColR (Francis et al., 2017), AgtS-AgtR (Francis et al., 2017), PprA–PprB (de Bentzmann et al., 2012), CbrA–CbrB (Yeung et al., 2011)	BvgAS (Roy et al., 1989)
Colonization of lower respiratory tract	SagS (Petrova and Sauer, 2011), HptB-HsbR (Bhuwan et al., 2012; Chambonnier et al., 2016), RocS2-RocA2 (Sivaneson et al., 2011), RoxS-RoxR (Hurley et al., 2010)	PlrSR (Bone et al., 2017)
Intracellular survival	GacS-GacA (Francis et al., 2017), RoxS-RoxR (Hurley et al., 2010),	RisAS (Jungnitz et al., 1998)
Acute/chronic switch	GacS-GacA (Bhagirath et al., 2017), CpxA-CpxR (Tian et al., 2016), KinA-AlgB (Chand et al., 2011, 2012)	
Quorum sensing dependent regulation	GacS-GacA (Francis et al., 2017), PhoR–PhoB (Bielecki et al., 2015), ParS-ParR (Wang et al., 2013), CzcS–CzcR (Sionov and Steinberg, 2022)	Unknown
Biofilm formation and maintenance	BfiS-BfiR (Petrova and Sauer, 2009), SagS (Petrova and Sauer, 2011), LadS (Chambonnier et al., 2016), RetS (Moscoso et al., 2011), PvrS-PvrR (Mikkelsen et al., 2013), RcsC-RcsB (Mikkelsen et al., 2013), ErcS, ErdR (Beaudoin et al., 2012), FimS (AlgZ)-AlgR (Okkotsu et al., 2014), PhoQ–PhoP (McPhee et al., 2006), CreC–CreB (Zamorano et al., 2014), BqsS-BqrR (Guragain et al., 2016), WspE–WspR (Huangyutitham et al., 2013), NarX–NarL (Benkert et al., 2008), BfmS-BfmR (Petrova and Sauer, 2009), PilS–PilR (Overhage et al., 2007), MifS-MifR (Tatke et al., 2015; Sarwar et al., 2020)	
Metal acquisition and resistance	PirR–PirS (Vasil and Ochsner, 1999; Francis et al., 2017; Mund et al., 2017), CzcS–CzcR (Dieppois et al., 2012), BqsS-BqrR (Guragain et al., 2016), PfeS–PfeR (Dean et al., 1996), CopS–CopR (Caille et al., 2007)	
Stress response	NtrB-NtrC (Li and Lu, 2007), CzcS–CzcR (Dieppois et al., 2012), BqsS-BqrR (Guragain et al., 2016), PfeS–PfeR (Dean et al., 1996), AmgS-AmgR (Lau et al., 2015)	

### Adapting to colonize

After bacteria sense inflammatory signals and alter gene expression to respond to external stimuli, the battle with the host immune response begins. Now is when the colonization and grow really start, spreading to new tissues and escaping immune clearance. *P. aeruginosa* and *Bordetella* spp. have regulatory systems for controlling the switch between acute and persistent infection. We will discuss three main aspects of the colonization process related with bacterial motility and adhesion, bacterial toxins, and finally we will focus on one of the most powerful bacterial immune-suppressive mechanisms, secretion systems.

#### Motility

Motility is a very important characteristic of bacterial pathogens. Flagella not only increase attachment that leads to biofilm formation, but flagella are also a very strong activator of the Toll-Receptor 5 (TLR5) (López-Boado et al., 2005) which will lead to a strong activation of the host immune response. Due to their great antigenicity surface molecules in bacteria must be tightly regulated to escape immune recognition. In *Pseudomonas* spp. an initial step in host interaction is mediated by polar pili that activate the host NF-κB signaling pathway promoting pro-inflammatory responses (Sadikot et al., 2005). Contact between the pili of *P. aeruginosa* induce the expression of virulence genes (Laventie et al., 2019). Overall, *P. aeruginosa* utilizes pili to manipulate host immune responses. Similarly, *Bordetella* spp. pili modulate host immunity and possess antigenic activity (Lee et al., 1986). In *Bordetella* spp. pili are mostly known as fimbriae (Melvin et al., 2014; Scheller and Cotter, 2015). They are major adhesins that promote biofilm formation and attachment of *Bordetella* spp. (Cotter et al., 1998; Irie et al., 2004; Mishra et al., 2005; Ulett et al., 2007; Lasaro et al., 2009; Wurpel et al., 2014; Scavone et al., 2016). Fimbriae-null mutants fail to persist in the mouse trachea due to impaired initial colonization (Mattoo et al., 2000). Overall, in both bacteria pili/fimbriae are critical for adhesion and initial colonization.

Another mechanism that facilitates motility is flagella. *P. aeruginosa* expresses polar flagella, which are necessary for motility and binding to TLR5, resulting in activation of interleukin-8 (IL8) (Sadikot et al., 2005). Flagella motility is common in *Pseudomonas aeruginosa* but interestingly clinical isolates present highly variable flagella motility and even in contact with sputum from cystic fibrosis patients, *P. aeruginosa* downregulates flagella expression (Lavoie et al., 2011), possibly to reduce the activation of inflammatory responses in the host. In *P. aeruginosa*, flagella is critical to trigger neutrophil traps activation (Floyd et al., 2016), a very powerful host pro-inflammatory response (Williams et al., 2020). Similar, in *B. bronchiseptica*, flagella initiate attachment (Nicholson et al., 2012; Cattelan et al., 2016) and play a critical role in modulating host immune responses (Akerley et al., 1995; Gestal et al., 2019a). Ectopic expression of flagella in *Bordetella* spp. promote rapid clearance from the respiratory tract (Akerley et al., 1995; Gestal et al., 2019a), possibly due to the activation of TLR5 (Gestal M. et al., 2019). Importantly, one of the hallmarks of *Bordetella* spp. infection is that during the virulence phase, *Bordetella* spp. downregulates flagella via Bvg TCS (Stibitz et al., 1988; Irie et al., 2004), as possible mechanism to evade immune response by avoiding TLR5 recognition.

#### Toxins: Suppressing host immune responses

After initial recognition escape, and while starting the colonization and grow processes, bacteria have to counteract immune defense utilizing several mechanisms, such as secreted toxins or direct delivery of toxins into host cells. Innate immunity plays an important role in both infections settings and neutrophils are in charge of clearance of early infection by both bacteria (Andreasen and Carbonetti, 2009; Rada, 2017). There are many different toxins that bacteria utilize to suppress host immune responses and neutrophil and other innate cells function and here we will focus on the most representative of each bacterium.

*Pseudomonas aeruginosa* synthesizes pigments that function as toxins to promote infection and colonization (Yabuuchi and Ohyama, 1972). Pigments such as pyocyanin inhibits phagocytosis by macrophages, induces apoptosis in neutrophils (Managò et al., 2015), and causes cellular damage, aiding in lung colonization and persistence, especially in cystic fibrosis patients (Lau et al., 2004). The antibacterial properties of pyocyanin have been thought to aid in eliminating competing bacteria, which allows *P. aeruginosa* to control many other pathogens and grow unimpeded in patients (Armstrong et al., 1971). In addition, pyocyanin modulates host airway epithelial cells by altering the expression of chemokines IL8 and RANTES and suppressing cilia beating (Denning et al., 1998; Look et al., 2005). Pyocyanin is not only related with neutrophil recruitment via IL8 and leukotriene B4 (Hall et al., 2016) but pyocyanin also suppresses the generation of efficient neutrophil responses by promoting the formation of neutrophils traps (Rada et al., 2013) and neutrophil death via aopotosis (Allen et al., 2005; Managò et al., 2015). A heat-labile hemolytic substance, which is suspected to be phospholipase C (PLC), is produced by *P. aeruginosa* (Sierra, 1960; Ostroff et al., 1989; McClure and Schiller, 1992). Amongst its functions, is to promote the activation of neutrophils (Jackon et al., 2000). The combination of hemolytic glycolipid and PLC has been hypothesized to produce extensive cytopathology in lung tissue amid *P. aeruginosa* pulmonary infection and contributes to *P. aeruginosa* colonization in the lungs (Holm et al., 1991). The extracellular polysaccharide Psl is expressed by non-mucoid *P. aeruginosa* strains and protects the pathogen by reducing neutrophil phagocytosis and limiting complement-mediated opsonization (Mishra et al., 2012). Another virulence factor identified to functionally inhibit host phagocytosis is the elastolytic metalloproteinase LasB, which cleaves host protease-activated receptors such as PAR2 (Dulon et al., 2005). The alkaline protease (AprA) of *P. aeruginosa* has been associated with bacterial virulence and is known to interfere with complement-mediated lysis of erythrocytes, while recent studies suggest it blocks complement activation via the classical and lectin pathways (Laarman et al., 2012).

Similar to *Pseudomonas, Bordetella* spp. also harbor toxins that block and suppress the generation of inflammatory responses. *Bordetella* harbor many mechanisms that can interfere with phagocytosis (Weingart and Weiss, 2000), but one of the most versatile toxins is the *Bordetella* spp. pertussis toxin (PT). PT is an AB5 toxin, that interferes with innate immunity by manipulating macrophages (Saukkonen et al., 1991; Carbonetti et al., 2003, 2007), neutrophils (Andreasen and Carbonetti, 2008, 2009), immune signals (Tonon et al., 2002; Wakatsuki et al., 2003; Andreasen et al., 2009), and T-cell responses (Chen et al., 2006; Tonon et al., 2006; Schneider et al., 2007). PT also impairs the generation of antibodies (Kirimanjeswara et al., 2005) and even affects metabolism such as glucose homeostasis by targeting G-protein coupled receptors (GPCRs) (Freyberg and Harvill, 2017). PT not only exerts its role on host immune responses, but it can modulate neuronal responses (Vega et al., 2020), which impacts not only disease pathogenesis (Murphy et al., 2020; Paramonov et al., 2020), but also future investigations and treatment (Zhou et al., 2020) of neurological disorders. Another powerful toxin of *Bordetella* spp. is the adenylate cyclase toxin (ACT), a pore forming toxin, which is one of the major immunomodulators of *Bordetella* spp. It is involved in manipulation of phagocytosis (Martín et al., 2015), suppression of antigen presenting cells (Fedele et al., 2017), blockade of neutrophil bactericidal activities (Cerny et al., 2017), modulation of macrophage differentiation (Cheung et al., 2008; Ahmad and Sebo, 2020), adhesion to epithelial cells (Angely et al., 2017), and T-cell differentiation (Paccani et al., 2008; Dunne et al., 2010; Svedova et al., 2016). *Bordetella* spp. also breaks down erythrocytes, one of the main mechanisms is via adenylate cyclase toxin (ACT) (Bemis and Plotkin, 1982; Vojtová et al., 2006). ACT also is responsible for the lysis of other immune cells including monocytes (Basler et al., 2006), macrophages (Hewlett et al., 2006), and even interferes with neutrophil trap formation (Eby et al., 2014).

**FIGURE 1:**
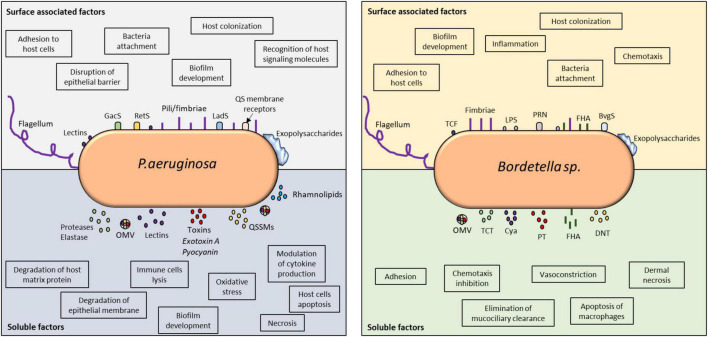
Virulence factors involved in immune detection and evasion by *P. aeruginosa* and *Bordetella* spp. Comparative analysis of the surface-associated and soluble virulence factors of these two microorganisms revealed the utilization of classical adhesins for cellular attachment, host colonization, and invasion. The impact of their virulence factors on host immune modulation can be found in the highlighted boxes. Both microorganisms utilize TCSs to detect host signals and modulate their behavior. In *P. aeruginosa*, QS receptors can also detect host molecules, such as hormones and cytokines. Their soluble factors, especially toxins and lysins, are vital in host invasion and immune evasion because they control inflammation and can kill immune cells. Biofilms produced by these microorganisms are highly controlled at the molecular level and responsible for persistent infection. QS, quorum sensing; OMV, outer membrane vesicle; QSSM, quorum sensing signaling molecule; PT, pertussis toxin; TCF, tracheal colonizing factor; Cya, adenylate cyclase; TCT, tracheal cytotoxin; DNT, dermonecrotic toxin; PRN, pertactin; FHA, filamentous hemagglutinin.

Overall, *P. aeruginosa* and *Bordetella* spp. have similar strategies that allow them to overcome host responses by utilizing similar toxin-mediated mechanisms to block, suppress, and manipulate host immunity (Figure 1).

#### Secretion systems

Secretions systems are one of the most powerful mechanisms to manipulate host immune cells (Coburn et al., 2007). Six secretion systems have been described in *P. aeruginosa* but their roles in infection are still being investigated. The Type 3 secretion system (T3SS) is associated with acute infection, the type 4 secretion system (T4SS) with biofilm development are involved in chronic infection (Zhu et al., 2016a,b), and the T6SS is important for QS signaling, host-pathogen communication (Gallique et al., 2017), and competition with other microbiota (Weyrich et al., 2014).

T3SS is critical for several aspects of the infection process of *Pseudomonas* spp. and *Bordetella* spp., including adherence and invasion of host cells (Ridley, 2006), intracellular survival (Rivera et al., 2019), cytotoxicity against several immune cells (Hauser, 2009; Bayram et al., 2020; Kamanova, 2020), and manipulation of immune cell signaling (Hauser, 2009, 2011). Vance et al. demonstrated that the *P. aeruginosa* T3SS is vital for bacterial survival when the infection reaches the blood, further indicating that is required to combat immune cells that *P. aeruginosa* might encounter in the bloodstream (Vance et al., 2005; Furukawa et al., 2006). There is mounting evidence that demonstrate that the T3SS in *P. aeruginosa*, and related *Pseudomonas* spp. strains, is regulated via QS by an intricate network of bacterial signals that respond to host inflammatory cues (Pena et al., 2019; O’Malley and Anderson, 2021).

In *B. bronchiseptica*, the T3SS consists of approximately 30 proteins encoded by the *bsc* (Kamanova, 2020) locus and it regulation is tighten to the sigma factor *brpL* (Moon et al., 2017), also known as *btrS.* Expression of the T3SS in *Bordetella* spp. also respond to external cues, such as CO_2_ (Hester et al., 2012), iron (Brickman et al., 2011; Kurushima et al., 2012b), or blood (Gestal et al., 2018; Petráçková et al., 2020; Drzmisek et al., 2021); however, it is still not clear how the molecular network of regulators is connected. It was thought that the T3SS was regulated by the Bvg TCS (Nicholson, 2007; Moon et al., 2017); yet, recent evidence demonstrate that this is not correct. Instead the Bvg regulates *btrS*, which downstream controls the expression of the T3SS locus (Coutte et al., 2020). Nevertheless, these findings demonstrate that the regulation of the T3SS is more complex than only one regulator (Gutierrez et al., 2022), highlighting the importance of the meticulous regulated network of virulence factors. In *Bordetella* spp. the T3SS is critical for a successful colonization and persistence in the host as its role during immune suppression is critical. T3SS suppress the migration of antigen presenting cells (Skinner et al., 2005), induces macrophage and neutrophil apoptosis (Stockbauer et al., 2003), differentially activates inflammatory pathways (Yuk et al., 2000), and modifies other immune signals (Pilione and Harvill, 2006), all of which is mostly mediated by the secreted effector proteins, BteA and BopN (Bayram et al., 2020).

Secretion systems in many bacteria are one of the greatest bacterial immunomodulator, it is highly conserved amongst different bacterial species, and in fact, they are required for acute and/or chronic infection for many microbes. Albeit there is a lot of research demonstrating the critical roles of several secretion systems in bacterial interactions with host immune responses, there are still new roles and mechanisms that are still not fully understood. The in-depth understanding of the molecular mechanisms by which T3SS suppresses host immune responses might present a new avenue for therapeutic development.

### Orchestration of the bacterial responses

Bacteria can very efficiently sense and respond to host cues some of which will provide a significant advantage during the infection settings. Bacterial ability to hijack host metabolism (Freyberg and Harvill, 2017) to scavenge nutrients (Murdoch and Skaar, 2022) is a very important feature of bacterial pathogens. Iron (Fe) is required by virtually all vertebrates but also bacterial pathogens, therefore, following invasion by pathogenic bacteria, the host limits bacterial access to Fe through a systemic reprogramming of Fe homeostasis by sequestering Fe in macrophages, hepatocytes, and enterocytes, while simultaneously reducing uptake of Fe from the diet. Bacterial pathogens can scavenge Fe through concerted action of secreted siderophores, uptake of host haem or Fe-containing molecules (transferrin and calprotectin) and uptake of ferrous Fe (Murdoch and Skaar, 2022). *Pseudomonas sp*. secrete haemolysins that integrate into erythrocyte membranes and result in osmotic lysis (Martins et al., 2016). Bacterial haem acquisition permits bacterial survival amidst the presence of host Fe-chelating molecules. In the presence of the Fe-sequestering protein calprotectin, haem availability is essential for the survival of *P. aeruginosa*, highlighting the importance of haem as an Fe source within the host. The host counteracts bacterial haem scavenging using haptoglobin, which binds haemoglobin and reduces accessibility of haem while promoting clearance by host cells (Murdoch and Skaar, 2022). In *Bordetella* spp. iron is also a critical nutrient and several studies have focused on how *Bordetella* sense the iron and how it scavenges it. Via ACT toxin and other hemolysins, *Bordetella* spp. breaks down erythrocytes to have access to the iron. The iron starvation response is mediated by Fur (Brickman and Armstrong, 1995, 2018), which responds to the presence of cathecolamines (Brickman et al., 2011) or blood and serum (Gestal et al., 2018). But iron stress even promotes binding to the respiratory epithelia (Vidakovics et al., 2007) indicating that is not simply a nutritional exchange but also it is involved in the virulence and attachment.

Zinc (Zn) is essential to host immune function, and even mild zinc insufficiency leads to widespread defects in both innate and adaptive immunity, resulting in impaired clearance of pathogens. *P. aeruginosa* have developed zinc uptake systems (ZnuABC, HmtA, and ZrmABCD) which are regulated by QS and specific molecules, such as the zinc uptake regulator (Zur) (Wang et al., 2021). Although Zn uptake systems are not yet clearly understood, to ensure a successful infection, *P. aeruginosa* must adapt to the environment of zinc deficiency, and this can provide new ideas and methods for the development of new drugs targeting *P. aeruginosa* zinc uptake systems and corresponding treatments for infection (Wang et al., 2021). In *Bordetella* spp. MerR also responds to nutrients such as Zn (Kidd and Brown, 2003). However, recent research focus on understanding how *Bordetella* spp. can also modulate virulence in response to other host metabolites (Gonyar et al., 2019) including copper (Rivera-Millot et al., 2021; Roy et al., 2022), manganese (Čapek et al., 2021) or glumatate (Keidel et al., 2018).

In response to nutrients, hormones, and other host cues, bacteria can meticulously regulate the expression of the aforementioned virulence factors which are critical for the successful colonization and infection of the host. Flagella, toxins, and secretions systems allow bacteria to colonize and conquer the host, but how do bacteria regulate gene expression once the cue has been sensed by the TCS? To orchestrate how bacteria sense and respond to environmental cues, a system needs to be in place allowing for bacterial communication, this mechanism is known as quorum sensing (QS). QS is very well studied in various model organisms including, *Vibrio* spp. or *P. aeruginosa* and contrary in other pathogens, such as *Bordetella spp*., the lack of understanding of bacterial communication is noticeable. Importantly, QS is not the only mechanism that bacteria utilize to regulate and finely tune gene expression and amongst the classical bacterial stress response other mechanisms of regulation are included such as sigma factors, small RNAs, alarmones, and chaperones which we will briefly discuss below.

### Quorum sensing

*Pseudomonas aeruginosa* coordinate gene expression in a cell density-dependent manner via an intricate QS network with four intertwined QS systems: *las, rhl, pqs*, and *iqs* (Lee and Zhang, 2015; Schütz and Empting, 2018; Meng et al., 2020). Two acyl-homoserine-lactone circuits, LasI-LasR and RhlI-RhlR (Turkina and Vikström, 2019), that are required to activate many genes, including those encoding virulence factors (Kostylev et al., 2019). In *P. aeruginosa*, the role of QS during host interaction and inter-kingdom communication, has been widely studied and many papers and reviews in depth explore these aspects (Holban et al., 2014a; Turkina and Vikström, 2019). In fact, QS molecules themselves have immunomodulatory activity. *N*-(3-Oxododecanoyl)-l-Homoserine Lactone (3-Oxo-C12-HSL) reduces the production of proinflammatory cytokines such as IL-12 (Telford et al., 1998), inhibits T cell differentiation (Ritchie et al., 2005, 2007), and promote anti-inflammatory responses in several cells including macrophages (Coquant et al., 2020), indicating that QS molecules not only function in bacterial communication, but also in host-interactions.

Despite the obvious critical role of QS during the infectious process, in *Bordetella* spp. there is still no evidence of a similar mechanism (Irie et al., 2005). Nevertheless, *Bordetella* species exhibit key virulence behaviors associated with QS, such as biofilm development or preventing biofouling in membrane bioreactor systems suggesting that a QS-like mechanism in *Bordetella* spp. might be present (Ergön-Can et al., 2020).

### Sigma factors

After the TCS activates the signaling cascade downstream, sigma factors are one of the regulators that will be induced in order to facilitate bacterial adaptability to the stressor. Sigma (σ) factors are critical during bacterial stress response. They are dissociable units of prokaryotic RNA polymerase that direct the holoenzyme to recognize conserved DNA motifs to modulate gene expression (Boor, 2006). During infection, σ factors regulate virulence factors, including secretion systems and secreted proteins, in *P. aeruginosa* (Otero-Asman et al., 2020) and *Bordetella* spp. (Ahuja et al., 2016), which are critical during host immune-suppression.

The σ^ECF^ factor family are important signal-responsive regulatory proteins in *P. aeruginosa*. Most of the *P. aeruginosa* sigma factors belong to iron starvation (IS) responses which are essential during host colonization and dissemination of *P. aeruginosa* during infection (Damron et al., 2016). The second most abundant σ^ECF^ group in *P. aeruginosa* is the RpoE-like σ^ECF^ factors, which are activated in response to cell envelope stress and activate the expression of genes involve in stress mitigation and bacterial cell envelope integrity, ensuring pathogen survival (Potvin et al., 2008; Chevalier et al., 2019). σ^ECF^ factors are critical for host immunomodulation and successful colonization (Skopelja-Gardner et al., 2019).

Similarly, in *Bordetella* spp. there are many different sigma factors that respond to stress and that play a role during colonization such as RpoE (Hanawa et al., 2013; Barbier et al., 2017). The *Bordetella* spp. σ^ECF^ factor *btrS/brpL* has been the best studied (Mattoo et al., 2004). *brpL/btrS* has been known for its role coordinating expression of the type 3 secretion system (T3SS) in conjunction with an anti-sigma factor known as *btrA* (Ahuja et al., 2016; Nakamura et al., 2019). Moreover, *brpL/btrS* was shown to suppress host immune responses in a variety of ways to promote long-term infection and even re-infection (Gestal et al., 2019a, 2020, 2022). It is important to highlight protein sequence of RpoE shares up to 76% sequence homology between *Pseudomonas* spp. and *Bordetella* spp. Moreover, in *P. aeruginosa* sigma factors such as *rpoE*, are regulated by the GacAS system. Similarly, the *brpL/btrS*,of *Bordetella* spp., is regulated by the BvgAS two component systems aforementioned (Coutte et al., 2020).

Although there are commonalities on the regulation of sigma factors, the precise mechanism by which TCS modulate sima factors and the connections of sigma factors with other regulators are still not clear. But overall, sigma factors regulate many different genes including those critical for host-immune suppression.

### Small RNAs

Another regulatory signaling pathway that bacteria utilize to respond to the external stimuli are small RNAs (sRNAs). sRNAs are non-coding small molecules of RNA that inhibit the expression of the targeted genes via post-translational or chromatin-depending silencing (Zhang, 2009). These play critical roles in the regulation of important phenotypes, including virulence and host immunomodulation (Han and Lory, 2021).

In *P. aeruginosa*, more than half of the characterized sRNAs are dependent on or mediated by the Hfq chaperone, which we will discuss later, and or two component systems such as GacAS (Pusic et al., 2021). Examples of sRNAs include, RsmY and RsmZ, both of which are involved in the switch from the motile to the sessile mode of life, modulate the expression of the T3SS and type 6 secretion system (T6SS) (Mikkelsen et al., 2011) and, thus, the transition from acute to persistent infection (Li et al., 2017). Another example is the PrrF which is critical to maintain iron homeostasis during murine lung infection (Reinhart et al., 2017) which is a battle that needs to happen prior to bacterial colonization and during infection.

In *Bordetella* spp. most of the sRNAs identified in *B. pertussis in vitro* are regulated by Bvg (Hot et al., 2011) which is the master virulence regulon. Differences in their expression are dictated by the phase growth, with some expressed exclusively during the exponential phase (Hot et al., 2011). One example of these sRNAs is RgtA, that regulates expression of proteins related to glutamate transport, which appears to be critical for the adaptation to nutritional stress and the change to persistent infection in the upper respiratory tract (Keidel et al., 2018). It has also been shown that in *Bordetella* spp. sRNAs play a role during colonization and preliminary data revealed new sRNAs in *B. pertussis* isolated from the murine trachea 4 days post-infection suggests a different response *in vivo* (Hiramatsu et al., 2020).

The complexity of sRNAs networks is only starting to being uncovered in *Pseudomonas* spp. (Zhang et al., 2017) but in *Bordetella* spp. this field is still at early stages, and although there are groups that are focused on investigating these exciting regulatory mechanisms, there are still multiple opportunities to unravel this fascinated interactions between sRNAs, TCS and virulence factors.

### Alarmones

A less studied mechanism part of the bacteria stress response is the nucleotides guanosine tetraphosphate and pentaphosphate ((p)ppGpp). (p)ppGpp are molecular signals known as alarmones, that regulate gene transcription during stress responses such as signals of host immune resposnes (Pita et al., 2018). In other bacteria alarmones have an important role in allowing bacteria to overcome immune responses, such as tolerance to oxidative stress (Fritsch et al., 2020) and stress survival (Léger et al., 2021). In *P. aeruginosa*, alarmones modulate several aspects of tolerance to host responses (Khakimova et al., 2013), and pathogenesis and virulence, including QS, biofilms, and antibiotic tolerance (Khakimova et al., 2013; Diez et al., 2020) critical during acute infection (Xu et al., 2016). Similarly, in *Bordetella* spp. they are involved in the regulation of biofilm formation and maturation (Sugisaki et al., 2013; Cattelan et al., 2016), and up-regulation of the T3SS (Hanawa et al., 2016). The role of alarmones during virulence and pathogenesis is still not fully understood, but it is clear that they are an alternative or supplementary stress response crucial for the infectious process.

### Chaperones

Chaperones assist in conformational folding or unfolding, and the assembly or disassembly of macromolecular structures (Henderson et al., 2006). There is increasing evidence indicating that chaperones have immunomodulatory functions. In *P. aeruginosa*, chaperones play a role in pathogenesis, such as biofilms (Vallet et al., 2001), the T3SS (Shen et al., 2008), and mediating antibiotic resistance (Klein et al., 2019). Similarly, in *Bordetella* spp., chaperones play critical roles in the regulation of multiple virulence factors, such as the T3SS (Kurushima et al., 2012a), or FHA (Baud et al., 2011).

One of the best studied chaperone in bacteria and that is common to *P. aeruginosa* (Sonnleitner et al., 2006) and *Bordetella* spp. (Bibova et al., 2013) is Hfq. Although, Hfq is not involved in protein folding, it regulates gene expression, including the expression of sRNA. Hfq has not only being studied in these two model organisms but also in other pathogens where similar results were found. The high degree of conservation of *hfq*, combined with the overlapping function in multiple pathogens, suggest that the role of this chaperone might be critical during pathogenesis. In fact, *hfq* might have been critical during evolution (Mura et al., 2013).

## Biofilm development and chronic lung disease

Once colonization has occurred and undergo acute phase of disease, it is time to progress toward a chronic establishment of the infection. During the establishment of chronic infection, biofilms play important biological roles in bacterial pathogenesis, increasing survival under arduous conditions (Holban et al., 2014b), such as in the lungs of patients with cystic fibrosis (Vallet et al., 2004) and chronic obstructive pulmonary disease (COPD) (Garcia-Vidal et al., 2009; Versteegh, 2012; Hashemi et al., 2015; Blasi et al., 2020; Wilkinson et al., 2021), and leading to antibiotic resistance (Fux et al., 2005), providing a strong positive selective advantage for biofilm-forming bacteria. Thus, as compared to acute infections, which are characterized by an enhanced host immune response, chronic lung infections are characterized by a silenced immune response, combined with the formation of biofilms. Bacteria within the biofilm are protected from host immune effectors, antimicrobials, antibiotics. However they also have to adjust to an hypoxic, nutrient-deprived and acidic niche that further promotes immune depression (Arciola et al., 2018). Infectious biofilms determine abnormal activation of immune suppressive cells (i.e., regulatory T cells), while exhausting the antigen presenting cells (APCs), invaliding T cell priming, and waning antibody responses by inducing insufficient somatic hypermutations in B cells (Campbell et al., 2020; Heim et al., 2020).

In *P. aeruginosa* transition toward chronic lung infection consists of the following steps; (1) bacterial flagellar and pilus-dependent motility is reduced limiting the invasion capability, in the host recognition and phagocytosis are dampened; (2) in bacteria there is an increased expression of T3SS and T6SS which modulates interactions with host cells, but also PldA and PldB correlated to bacterial internalization in non-phagocytic cells while repressing the expression of regulatory molecules (RetS/GacS, cyclic-di-GMP); (3) bacteria have impaired production of secreted proteases (i.e., LasA, LasB, AprA, protease (4) leading to decreased host recognition and reduced host tissue destruction; (5) bacteria increase exopolysaccharides production, including alginate, Psl, Pel (Leid et al., 2005; Colvin et al., 2011; Sønderholm et al., 2017), and extracellular adhesins, such as CdrA, which promote bacterial aggregation and biofilm formation (Reichhardt et al., 2020); and finally (6) bacteria undergo lipopolysaccharide (LPS) modification, such as mutations in lipid A or loss of antigen O, leading to immune evasion (Faure et al., 2018).

In whooping cough *Bordetella pertussis* infections are not associated with a classical chronic infection, however *Bordetella* can develop biofilms and persist in the trachea and lungs. In *Bordetella* spp. biofilms, extracellular DNA (eDNA) plays a critical role (Holban et al., 2016), together with virulence factors associated with motility, such as flagella and fimbriae, or filamentous hemagglutinin (Serra et al., 2011), are pivotal to initial attachment and microcolony development (de Kievit, 2009; Holubova et al., 2022). In *Bordetella* spp., similarly, exopolysaccharides (Sloan et al., 2007) and adhesins are critical for initial attachment and aggregation, whereas eDNA promotes the formation and stability of three-dimensional biofilm structures (Cattelan et al., 2016). Although there is still debate about the clinical relevance of biofilms during *Bordetella* spp. infection, biofilms have been identified in patients with whooping cough, and clinical *B. pertussis* strains tend to produce more robust biofilms than lab-adapted strains (Arnal et al., 2015; Cattelan et al., 2017). This suggest that the reduced biofilm formation ability identified in lab strains can be a consequence of adaptation to the laboratory environment and an effect of passaging without a mammalian host.

**FIGURE 2:**
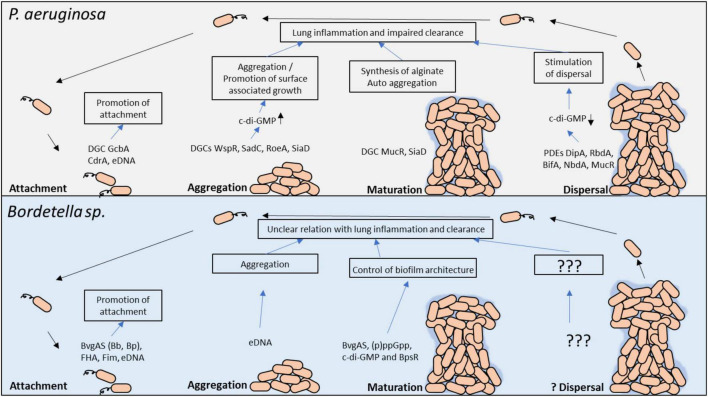
Molecular regulators in *P. aeruginosa* and *Bordetella* spp. biofilm development and their potential role in persistent lung infection. Each stage of biofilm development and the main molecular modulators are shown. The gray box above highlights the key factors involved in *P. aeruginosa* biofilm formation, whereas the light blue box below highlights the known factors in *Bordetella* spp. biofilm regulation. c-di-GMP, cyclic dimeric guanosine monophosphate; PDE, phosphodiesterase; DGC, diguanylate cyclase; eDNA, extracellular DNA.

It is clear that both bacteria produce biofilm however the molecular signaling mechanisms that trigger and regulate biofilm formation in response to external cues is not as well define and understood for *Bordetella* spp. as it is for *P. aeruginosa* (Figure 2). We propose that using the knowledge of biofilm gain from *Pseudomonas spp.* research can guide us on the investigations that can further clarify the role of *Bordetella* spp. biofilm in disease progression and clinical manifestation in patients with chronic lung disease or neonates, in whom biofilm can be fatal.

## Signaling applied to therapeutic development of lung co-infections

Nascent findings on the interactions between *P. aeruginosa* and *B. bronchiseptica* demonstrate that rhamnolipids from *P. aeruginosa* have the ability to successfully disrupt *B. bronchiseptica* biofilms, without antibacterial activity against planktonic *B. bronchiseptica* (Irie et al., 2005), suggesting a sensing/signaling mechanism between these two species. Interestingly, there is no molecular signatures that can relate to quorum sensing mechanisms in *Bordetella* spp. but the fact that biofilms are disrupted while planktonic bacteria are still viable, indicates that there must be a mechanism that allows *Bordetella* spp. to very tightly coordinate specific responses against these *P. aeruginosa* molecules (Irie et al., 2005). These findings are further supported by the previous findings that *Bordetella* spp. are able to respond to host cues such as CO_2_ (Hester et al., 2012), iron (Fe) (Brickman et al., 2011), blood, or serum (Gestal et al., 2018). Recently, immunomodulatory therapies are becoming cutting-edge research for not only for bacterial treatment but also as anti-biofilm strategies. Some therapies are decreasing lung pathology and reducing the detrimental effects caused by infection (Ernst et al., 2021; Gallop et al., 2021). Some research is focusing on enhancing the performance of innate immune cells such as neutrophils and macrophages. However, these therapies fail to demonstrate anti-biofilm effects, decreasing the enthusiasm. Next generation of infectious immunotherapy may be catalyzed by inducing targeting neutralizing antibodies and long-lasting memory responses against biofilms. However, there are several limitations on the design of adaptive immunity modulatory strategies focusing on biofilm eradication, and some of these limitations including the poorly immunogenic bacterial associated antigens in the biofilm fortress, which would result in deficient antigen presentation and suppressing T cell priming and activation.

## Concluding remarks and future perspectives

Bacteria have a very finely tune network of regulators that become critical during the battle with the host. In order to successfully colonize and cause long term infection, bacteria need to very precisely regulate expression of various immunomodulatory/virulence factors that suppress host immune response in a variety of ways. Understanding the molecular mechanisms underlying this fine regulation, can provide novel avenues for vaccine (Gestal et al., 2019b,a; Gestal M. et al., 2019) and therapeutic (Gestal M. et al., 2019) development. However, our current models have limitations and until now most of our investigations have been done *in vitro*. Combining the molecular knowledge available in *P. aeruginosa* and animal model of *Bordetella* spp. we can investigate the molecular mechanisms by which pathogens suppress host immune responses. These interesting and novel areas would shed light on the pathogenesis of bacterial communities within the lungs and provide knowledge for the development of efficacious therapeutic targets.

## Author contributions

AH and MG wrote the initial draft, edited and approved the manuscript for submission. CG wrote, edited and approved the manuscript for submission. All authors contributed to the article and approved the submitted version.
